# Nano- and microstructured materials for in vitro studies of the physiology of vascular cells

**DOI:** 10.3762/bjnano.7.155

**Published:** 2016-11-08

**Authors:** Alexandra M Greiner, Adria Sales, Hao Chen, Sarah A Biela, Dieter Kaufmann, Ralf Kemkemer

**Affiliations:** 1 Karlsruhe Institute of Technology (KIT), Institute of Zoology, Department of Cell and Neurobiology, Haid-und-Neu-Strasse 9, 76131 Karlsruhe, Germany; 2 now at: Pforzheim University, School of Engineering, Tiefenbronner Strasse 65, 75175 Pforzheim, Germany; 3 Max Planck Institute for Intelligent Systems, Department of New Materials and Biosystems, Heisenbergstrasse 3, 70569 Stuttgart, Germany; 4 Universitätsklinikum Ulm, Institut für Humangenetik, Albert Einstein Allee 11, 89070 Ulm, Germany; 5 Reutlingen University, Faculty of Applied Chemistry, Alteburgstrasse 150, 72762 Reutlingen, Germany

**Keywords:** fabrication methods, materials selection, nano- and micro-topography, vascular endothelial cells, vascular smooth muscle cells

## Abstract

The extracellular environment of vascular cells in vivo is complex in its chemical composition, physical properties, and architecture. Consequently, it has been a great challenge to study vascular cell responses in vitro, either to understand their interaction with their native environment or to investigate their interaction with artificial structures such as implant surfaces. New procedures and techniques from materials science to fabricate bio-scaffolds and surfaces have enabled novel studies of vascular cell responses under well-defined, controllable culture conditions. These advancements are paving the way for a deeper understanding of vascular cell biology and materials–cell interaction. Here, we review previous work focusing on the interaction of vascular smooth muscle cells (SMCs) and endothelial cells (ECs) with materials having micro- and nanostructured surfaces. We summarize fabrication techniques for surface topographies, materials, geometries, biochemical functionalization, and mechanical properties of such materials. Furthermore, various studies on vascular cell behavior and their biological responses to micro- and nanostructured surfaces are reviewed. Emphasis is given to studies of cell morphology and motility, cell proliferation, the cytoskeleton and cell-matrix adhesions, and signal transduction pathways of vascular cells. We finalize with a short outlook on potential interesting future studies.

## Introduction

**Figure 1:**
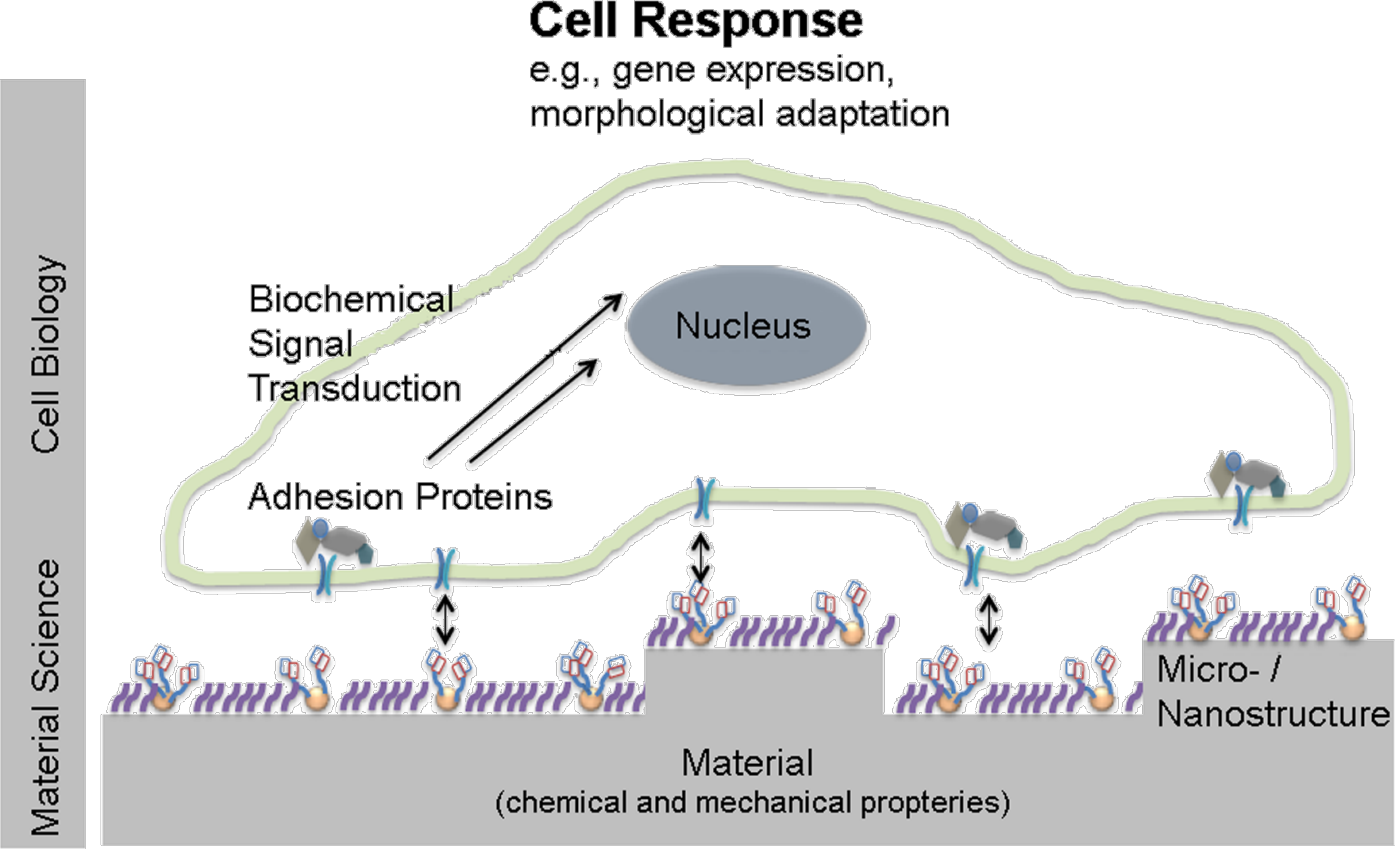
Schematic illustration of cell interaction with a micro- and nanostructured biofunctionalized surface and the two relevant size scales. The surface is structured with a micropattern, e.g., grooves or pits at the size of micrometers or below, corresponding to the size of cells. On a much smaller size scale, adhesive receptors interact with small surface features in the nanometer range. This interaction is schematically illustrated by nanoparticles that are functionalized with adhesive peptides. Cells interact with their extracellular environment by binding to cell adhesion-mediating molecules with their cell adhesion protein machinery. The surface topography and other characteristics such as the mechanical stiffness may lead to different availability of cell adhesion-mediating molecules and also require a deformation of cellular structures such as the cytoskeleton, adhesion sites or the membrane. All these interactions may transmit extracellular signals further into the cell yielding in a biological cell response. Typical examples might be changes in cell alignment, elongation, migration direction and gene expression.

**Figure 2:**
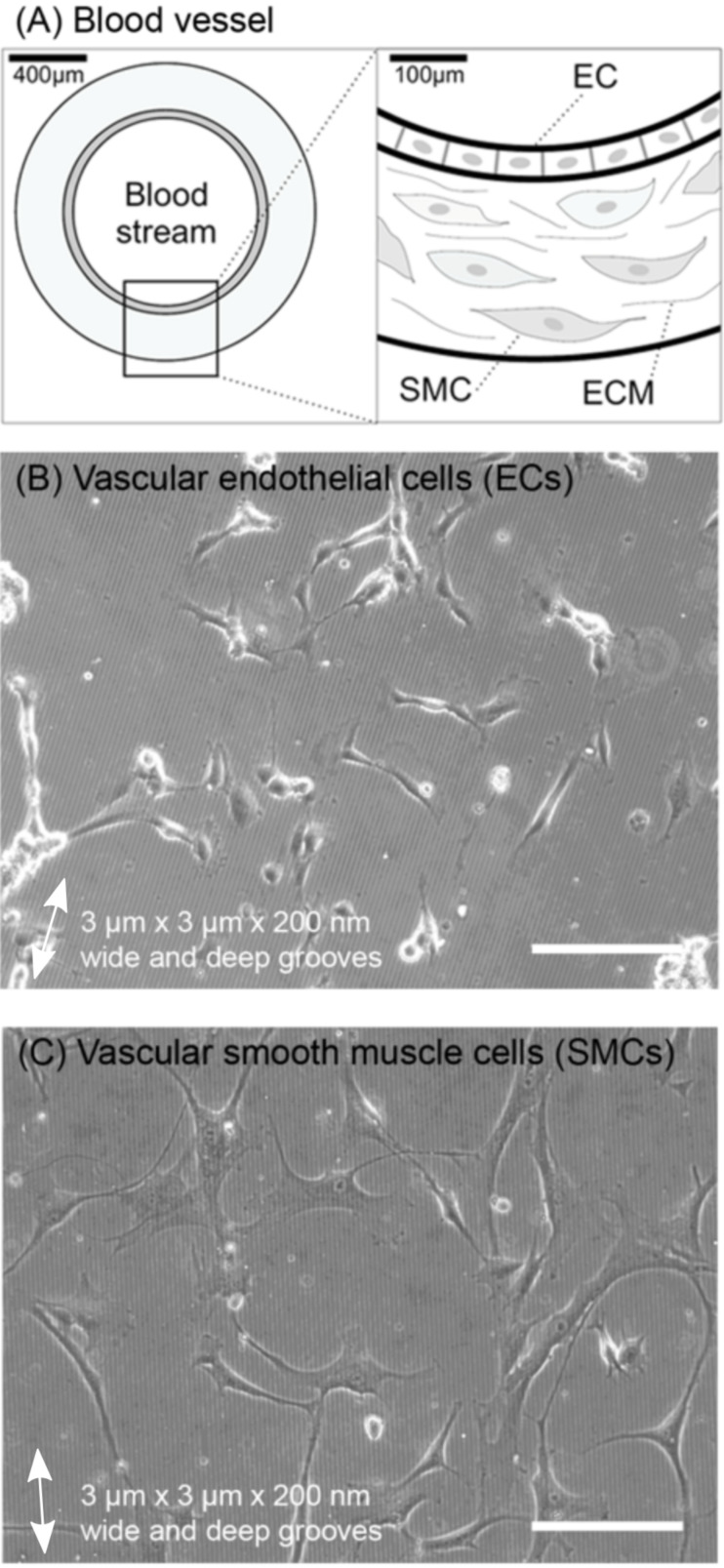
(A) Scheme of a blood vessel. Vascular endothelial cells (EC) form the inner dense cell layer of the blood vessel (endothelium) and are in direct contact with the blood. Vascular smooth muscle cells (SMC) build up a thicker outer layer surrounding the inner endothelium. SMCs are embedded within the extracellular matrix (ECM). (B,C) Morphology of (B) vascular ECs and (C) vascular SMCs cultured on fibronectin-coated, microstructured silicon-based polymeric substrates with 3 µm wide and 200 nm deep grooves (phase-contrast images, scale bar: 200 µm. Double white arrows indicate microgrooves direction).

**Figure 3:**
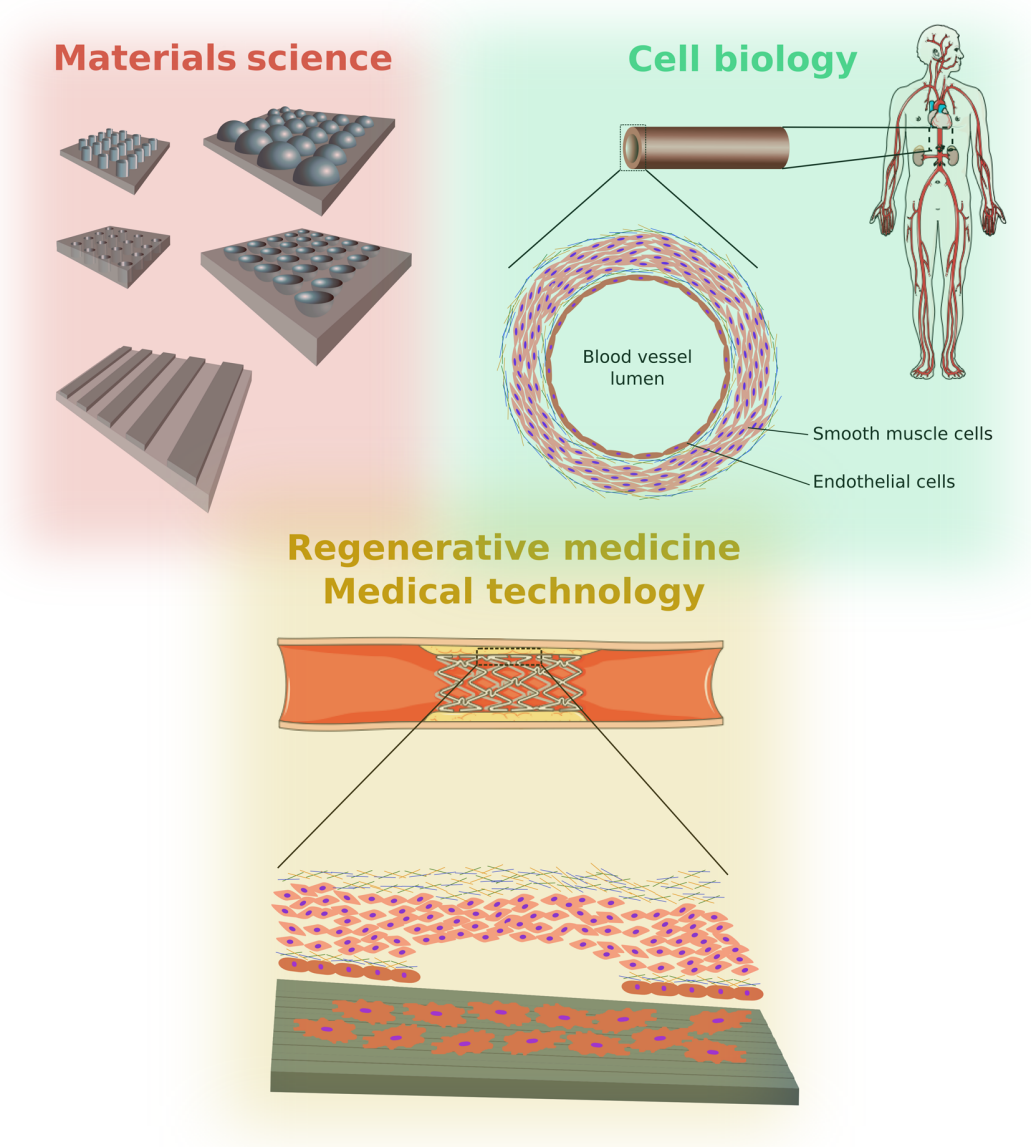
Materials science provides tools to create surface topographies with different geometries and sizes ranging from the nanoscale to the microscale. These topographies mimic in vivo environments in order to study biological processes and to develop new medical implants that are able to control cell behavior in vivo. Research of the reactions to vascular cells to surface topographies will enable the development of micro/nanostructured stents in order to improve wound healing and to control cell proliferation, thus avoiding the re-occlusion of the blood vessel (restenosis). The image of the human body, as well as the cross-sectional view of a stent inside a blood vessel have both been adapted from the Powerpoint Image Bank of the Servier Medical Art collection [1] under the CC BY 3.0 licence, copyright 2016 Les Laboratoires Servier.

Cells adhering to biomaterials are influenced by the surface topography, the surface chemistry and the mechanical properties of the substrate (Figure 1). In particular, the influence of the surface topography on cell behavior has been widely studied, with the motivation to understand the complex cell–substrate interactions and to transfer that knowledge to the design of implant surfaces. This review summarizes and discusses model studies with a special emphasis on the fabrication of substrates with well-defined nano- and microstructured surfaces for in vitro studies with vascular cells (Figure 1). Vascular endothelial cells (ECs) and smooth muscle cells (SMCs) are two vascular cell types forming blood vessels (Figure 2). They are key players in cardiovascular diseases and are the cells getting in immediate contact with many cardio-vascular medical devices such as stents. The integrity of the endothelia cell layer is essential for avoiding thrombosis. The detailed understanding of the responses of ECs and SMCs to different physical and chemical properties of an adhesive surface may lead to a better understanding of their biology and the origin of vascular diseases and malfunctions. Additionally, such knowledge will be supportive for the adequate and successful design and development of medical implants, e.g., stents. Thus, in vitro studies using modified artificial surfaces to induce biological responses in these cells are an important experimental model in vascular cell biology and biomaterial research (Figure 3).

In this review, we provide an overview of materials and important micro- and nanofabrication techniques that have been used for the fabrication of appropriate substrates for in vitro studies with vascular cells. We give a brief overview over possible surface structure geometries, mention compounds and methods for surface biofunctionalization and present the importance of the mechanical characteristics of cell-study relevant micro/nanostructured surfaces. In the last section of this review, we evaluate and summarize reports about studies of vascular cells interaction with micro/nanostructured surfaces.

## Review

### 1 Fabrication of micro- and nanopatterned substrates for cell biology studies

The development of micro- and nanofabrication techniques has permitted the manufacturing of precise surface topographies of materials surfaces. Samples with specific surface features haven been widely used for in vitro cell biology studies either to manipulate cell adhesion and resulting cell responses or to give surfaces sensor capabilities [2–11]. The topographies either mimic typical shapes and feature sizes found in the natural environment of cells or expose them to rather artificial often well-ordered geometries. Considering the length scales of interaction, either on the molecular size of adhesion proteins and their ligands, or on the size of cells, the effective structuring of surfaces with nanometer to micrometer precision is required. In this section, we review the currently available most common techniques and materials applied for the fabrication of appropriate micro/nanotopographies. We define micro/nanostructured substrates as materials having fabricated surface structures in all three dimensions and, consequently, not having a planar surface.

**Figure 4:**
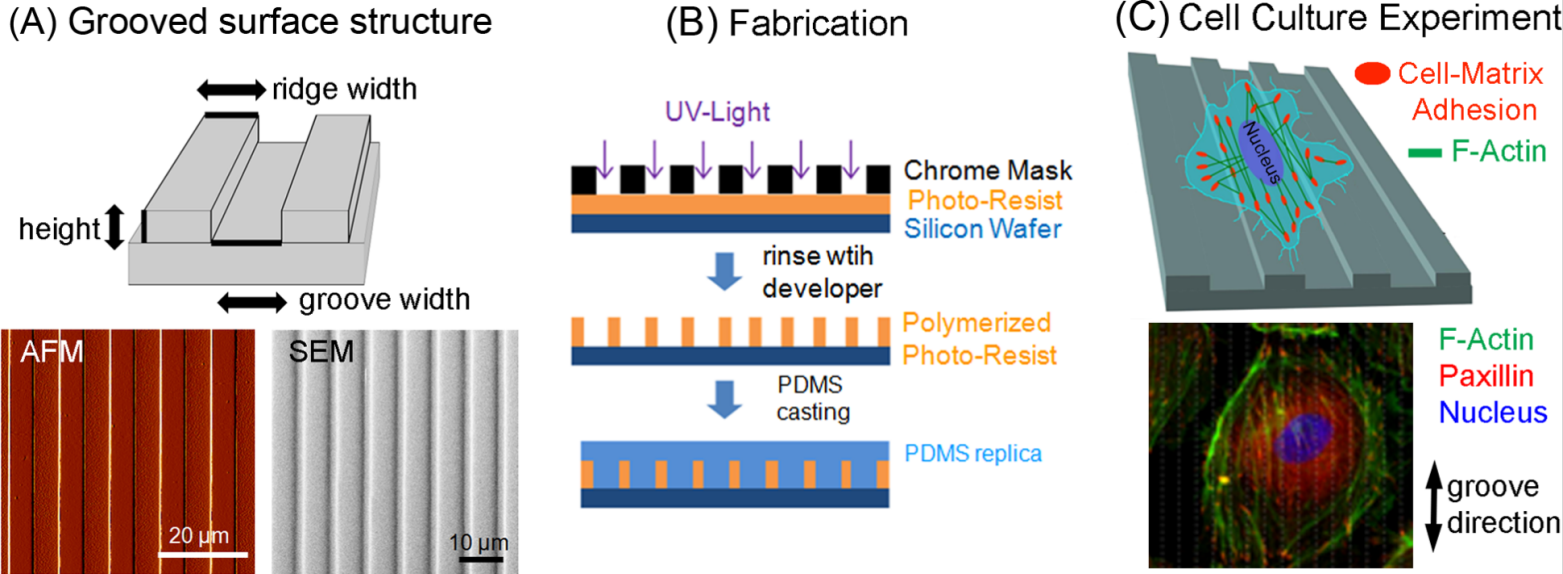
(A) The microgrooved PDMS replica is properly characterized through atomic force microscopy (AFM) and scanning electron microscopy (SEM) to confirm the dimensions of the groove structure are identical to the master structure. Subsequently the microgroved PDMS structure can be homogenously (or selectively) functionalized with cell adhesion-mediating biomolecules (such as the extracellular matrix protein fibronectin). (B) Flow chart of the fabrication of microgroove structured poly(dimethylsiloxane) (PDMS) substrates following the principles of soft lithography. A sandwich made of chrome mask, photoresist, and silicon wafer is illuminated by UV light to induce photo-resist polymerization. The polymerized photoresist forms structures on the silicon wafer as a master structure for the PDMS replica. The PDMS is molded on the master structure and is peeled off after polymerization resulting in a PDMS replicate with microgrooved structures. (C) Vascular cells are cultured on the bio-functionalized microgrooved PDMS substrates. The direction of the grooves is indicated by the black arrows. The F-actin cytoskeleton (green), the cell nucleus (blue) and the cell–matrix adhesion sites (paxillin; red) can be visualized by immunochemistry or staining with specific dyes through fluorescence microscopy.

The number of possible architectures of micro/nanostructured substrates is huge and it is often difficult to keep track with all varieties. However, common architectures are pores, gratings, wells, pits, cones, posts, pillars, grooves (Figure 4A) or mesh-like structures that can either be organized in a regular or irregular manner. For a systematic overview of the information presented in this section and about the fabrication techniques and the selection of materials for micro/nanostructured substrates as well as common geometries please refer to Table 1.

**Table 1:** Overview of in vitro studies of vascular cell responses to micro/nanostructured surface features. Various materials, fabrications methods and geometries (dimensions) are employed and different biological readouts using vascular smooth muscle cells (SMCs) and/or vascular endothelial cells (ECs) are supplied.

geometry	feature size^a^	material	fabrication method	cell type	biological response	ref.

grooves	*d* = 50–200 nm *w* = 2–10 µm	PDMS^b^	photolithography; soft lithography	ECs, SMCs	cell orientation and migration along grooves; enhanced cell elongation	[12]
grooves	*d* = 0.2–5 µm *w* = 3.5 µm	PDMS	photolithography; reactive ion etching; soft lithography	ECs	cell body, actin and focal adhesion orientation along grooves; proliferation is not influenced	[13]
grooves	*d* = 10 µm *w* = 30 µm	PDMS	photolithography; soft lithography	ECs	cell body, actin and focal adhesion orientation along grooves; changes in gene expression	[14]
grooves	*d* < 1 µm *w* < 1 µm	PDMS	surface cracking	SMCs	increased focal adhesion size along grooves	[15]
grooves	*d* = 350 nm *w* = 350 nm	PDMS, PMMA^c^	nano-imprinting; soft lithography	SMCs	increased cell and nucleus elongation; cell body and actin fiber orientation along grooves; reduced proliferation	[16]
grooves	*d* = 1.5 or 5 µm *w*(ridge) = 5, 10, 20 µm *w*(groove) = 5µm	PDMS	photolithography; reactive ion etching; soft lithography	ECs	cell body and nucleus orientation along grooves	[17]
grooves	*d* = 450 nm pitch = 2.5–4.5 µm	PGS^d^	photolithography; plasma etching; soft lithography	ECs	cell alignment along grooves; decreased circularity	[18]
grooves	*d* = 0.1–1 µm *w* = 1 µm	COC^e^	nano-imprint lithography	ECs	enhanced cell adhesion on shallow grooves; variations in focal adhesion composition on different grooves	[19]
grooves	*d* = 0.1–2 µm *w* = 1–5 µm	COC	nano-imprint lithography	ECs	early onset of cell spreading induced by grooves	[20]
grooves	*d* ≈ 200 nm *w* = 750 nm to 100 µm	Ti	photolithography; plasma dry etching	ECs	cell alignment along grooves; increased cell elongation; higher cell density (on grooves with *w* < 10 µm)	[16]
grooves	*d* = 11 µm *w*(groove) = 20–60 µm *w*(ridge) = 10 µm	PDMS	plasma etching; soft lithography	SMCs	increased alignment of cell body, actin fibers and nucleus on narrow grooves	[21]
grooves	*d* = 2.8 µm *w* = 10 µm	PDMS	photolithography; soft lithography	SMCs	enhanced cell elongation and orientation along grooves; decreased cell area and cell body/nucleus ratio; reduced proliferation	[22]
grooves	*d* = 500 nm *w* = 22–80 µm with micro- and nanoroughness	PDMS, Ti	electron beam lithography; physical vapor deposition; soft lithography	ECs	enhanced cell adhesion, elongation and increased cell density on nanorough areas	[23]
grooves	*w* = 20, 50, 80 µm *d* = 5 and 12 µm	PDMS	photolithography; soft lithography	SMCs	enhanced cell/nucleus aspect ratio and cell alignment; ECM remodeling	[24]
grooves	*w* = 350, 700, 1050 nm d: 500 nm	PLGA^f^	thermal imprinting	ECs	enhanced adhesion strength; increased cell alignment along grooves	[25]
grooves	*w*(ridge) = 600 nm *w*(groove) = 1200 nm *d* = 600 nm	PDMS	photolithography; soft lithography	ECs	increased cell elongation, alignment and migration along grooves; reduced cell proliferation	[26]

ripples	*h* = 25–100 nm	PET^g^	UV lithography	ECs	nuclear β-catenin accumulation (proliferative phenotype)	[27]
ripples	*w* = 620 nm *d* = 100 nm *h* = 15–600 nm	nitinol	laser lithography	ECs	increased cell orientation along the structures	[28]

convex hemi-spheres	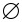 = 190–950 nm *h*: 5–396 nm spacing = 195–957 nm	PLGA	soft lithography	ECs	increased cell adhesion	[29]

pores	*d* = 20 nm, *d* = 200 nm	alumina membranes	commercially available	SMCs	enhanced cell proliferation and gene expression (on 200 nm pits)	[30]

tubes	*l* = 1 µm 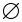 = 30 nm	TiO_2_	anodization	ECs, SMCs	enhanced proliferation of ECs; decreased proliferation of SMCs	[31]
tubes	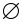 = 15–100 nm	TiO_2_	anodization	ECs	increased cell adhesion, proliferation and motility (on nanotubes with 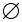 = 15 nm)	[32]
tubes	*l* > 400 nm 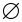 = 30 nm	TiO_2_	anodization	ECs, SMCs	increased proliferation; enhanced filopodia formation; increased cell elongation	[33]
tubes	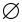 = 22–250 nm thickness = 7–30 nm	TiO_2_	anodization	ECs, SMCs	decreased cell proliferation; increased expression of SMC α-actin	[31]

pillars	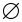 = 1–5.6 µm *h* = 1–8 µm	SiO_2_, PDMS	photolithography; reactive ion etching; soft lithography	ECs	decreased cell adhesion and spreading (on SiO_2_ pillars with *h* > 3 µm); enhanced cell alignment and elongation (on PDMS pillars)	[34]
pyramids	*h* = 50–1850 nm	Si	wet chemical etching	ECs	reduced cell migration; decreased adhesion	[35]
cones	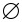 = 50 nm (at tip) *h* = 300–500 nm spacing: 150 nm	PEG-DMA^h^	Soft lithography	ECs	Increased cell adhesion	[36]
cones	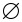 = 0.15 µm (at tip) h: 10 µm spacing: 6 µm	Silicon	Laser surface texturing	ECs	Increased cell spreading and adhesion	[37]

hills/bulges	*h* = 13–95 nm	PS/PBrS^i^	polymer demixing, spin coating	ECs	increased cell adhesion and spreading (on islands with *h* = 13 nm)	[38]
hills/bulges	*h* = 27 nm 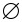 = 223 nm spacing = 1638 nm	PCL^j^/PEG	polymer demixing, spin coating	ECs	reduced cell adhesion and spreading	[39]
hills/bulges	*h* = 13–95 nm	PS/PBrS, PnBMA^k^/PS	polymer demixing, spin coating	ECs	increased cell adhesion (on islands with *h* = 13 and 18 nm)	[40]

random	surface roughness, 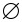 = 50 nm to 15 µm	PLGA, PCL, PU^l^, PDMS	chemical etching; soft lithography	SMCs	increased cell adhesion and proliferation	[41–42]
random	nanoroughness, 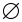 = 7–21 nm	SiO_2_	coating	ECs	decreased cell adhesion, spreading and proliferation	[43]
random	mesh composed of fibers with 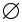 = 13 µm	PGA^m^	surface hydrolysis	SMCs	increased cell adhesion and proliferation	[44]
random	surface roughness in the sub-micrometer to nanometer range	PU, PLGA	chemical etching	SMCs	increased adhesion and proliferation	[41–42]
random	nanoroughness, about 11 nm	PCL	hot pressing	SMCs, ECs	increased cell adhesion and proliferation	[45]

^a^*d*: depth; *h*: height; *w*: width; 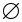: diameter; *l*: length; ^b^PDMS: poly(dimethylsiloxane); ^c^PMMA: poly(methyl methacrylate); ^d^PGS: poly(glycerol sebacate); ^e^COC: cyclic olefin copolymer; ^f^PLGA: poly(lactic-*co*-glycolic acid); ^g^PET: poly(ethylene terephthalate); ^h^PEG-DMA: poly(ethylene glycol) dimethacrylate; ^i^PS/PBrS = polystyrene/poly(4-bromostyrene); ^j^PCL: poly(caprolactone); ^k^PnBMA: poly(*n*-butyl methacrylate); ^l^PU = poly(ether urethane); ^m^PGA: poly(glycolic acid).

#### 1.1 Fabrication methods

In order to create tailored cell culture substrates with surface topographies established methods such as photolithography, electron- and focused-ion beam lithography, stereolithography, direct laser writing, and block co-polymer micellar nanolithography are applied. Based on the fabrication approach, fabrication techniques can be divided whether they follow a top-down approach or a bottom-up approach. In the first approach, an already existing bulk material is structured while in the latter approach single subunits are used to build up a structured substrate (e.g., by layer-by-layer technique). Depending on the pattern design procedure, fabrication techniques can be distinguished in either computer-assisted methods or methods without the aid of computers, such as methods using the self-organization of macromolecular systems. Computer-assisted methods, also known as solid free-form or rapid prototyping, initally require the design of a computer model with a special software. The second step is then the realization of the computer model with a specific fabrication system that can be grouped in either laser-based system, 3D printing setups, and nozzle-based settings [46–48].

We group these fabrication techniques by the size and spatial resolution of the surface features that can be achieved. Not every method is suitable for the production of desired surface feature size. In particular, some methods are not suited for structuring surface topographies in the nanometer range.

#### 1.2 Microfabrication techniques

Microfabrication techniques are mainly used to generate surface structures in the micrometer range, which is the size scale of cells. In order to give an overview of different microfabrication techniques, relevant examples for different approaches such as optical (photolithography [49]), mechanical (hot embossing [50] and surface cracking [51–52]) or chemical (replica molding [53–54], phase separation micromolding [55–57], gas-based techniques [55–57] and porogen-leaching methods [55]) are described. They often rely on the fabrication of a master with a designed surface topography that subsequently is replicated by a polymer. Most of these techniques have been extensively used and have allowed for novel types of experiments in cell biology for the last two decades [53–54].

Photolithography uses light, a photomask and a photosensitive material (photoresist) to create a pattern in the micrometer (or sub-micrometer) range (Figure 4B). The thickness of the layer of photoresist will determine the height of the structures. The pattern of the photomask will determine the later dimensions of a surface structure [49]. This method can be combined with other procedures such as physical or chemical vapor deposition where the height of the pattern can be further controlled by depositing a nanometrically controlled layer, often using metals [58]. The surface structures made by photolithography are typically further used as a master structure for further processing.

Hot embossing also replicates micro- and nanofeatures of master substrates. In that case, a thermoplastic material is pressed on the mold at a high temperature to form the topography of the features in the plastic. Similar to replica molding in soft lithography, features down to around 10 nm can be replicated. Like soft lithography hot embossing is a cheap method suitable for large-scale manufacturing of substrates [50]. In principle, it can be used with many thermoplastic polymers.

Another method for generating surface topographies is surface cracking. It provokes controlled cracks on a surface of a material and surface microstructures but also nanotopographies can be obtained by this method [51–52]. After surface modification (e.g., plasma treatment) of an elastomer like poly(dimethylsiloxane) (PDMS) strain can be applied on the substrate. Due to the rigidity and fragility of the layer formed on the top of the elastic PDMS substrate, an array of parallel cracks perpendicular to the strain direction will be formed. Depending on the strain, its direction, and its amplitude, cracks with different shapes can be formed. These cracks are typically few hundreds of nanometers deep and between ca. 100 nm and ca. 3 µm wide. One advantage of this technique is the possibility to change in situ the size of the cracks by modifying the strain, enabling the study of cell adaptation to dynamic changes of the substrate topography [51–52].

Replica molding is a soft lithography technique that uses an elastomeric soft material to replicate patterns (Figure 4B) [53–54]. With that method mainly micrometer-sized topographies are produced in the elastomer. Structures with high aspect ratios (height/lateral distance) are not easy to replicate with this method. Due to the relative simple procedure, soft lithography and related methods have been widely used in cell biology studies and are widely established as a standard tool [53,59–61]. The soft material is poured onto the surface with the desired pattern and let to polymerize. After polymerization, the replica is peeled off from the mold (Figure 4B). The lower limit of replica topographies will depend on the material used to replicate. With (PDMS), a commonly used elastomer, it was possible to replicate nanostructures of few tens of nanometers, for example (Figure 4B) [53] After further modifications, the elastomer substrate can be used for cell experiments.

Phase separation micromolding is an alternative, less common microfabrication technique for structured substrates. This technique consists of separating a polymer solution in two phases, typically by means of the addition of a non-solvent or a change in temperature. The phase containing the major concentration of the polymer solidifies forming the replicate of the surface topography [2,55,62].

Gas-based techniques [55–57,63] and porogen-leaching methods [55] process polymeric material and are also applied to fabricate cell culture substrates. However, the substrates resulting from these fabrication methods are in most cases (irregularly) porous, foam-like 3D structures rather than (symmetrical) surface-patterned substrates.

#### 1.3 Nanofabrication techniques

Nanofabrication techniques are mainly used to generate surface structures in the nanometer range. Similar to the previous section, relevant examples of nanofabrication techniques using different approaches, such as optical (nanometer-scale optical photolithography [49,58,64–65], nanoimprint lithography [66]), etching (focused-ion beam [67] and electron-beam nanolithography [68]), electrical (electrospinning [69–72]), mechanical (nanoskiving [46,73], nanoimprint lithography [66]) and colloidal (colloidal lithgraphy [74–75]) are given here.

Nanoscale optical photolithography takes advantage of optical superresolution, with which it is possible to go below the light diffraction limit, to perform photolithography with nanometer resolution. For example, using nano-antennas it was possible, by two-photon polymerization, to produce photoresist nanodots with diameters below 30 nm [64]. In a different work, a plasmon was used to pattern a photoresist layer by means of NSOM (near-field scanning optical microscopy). A lateral resolution of about 50 nm was achieved, with a fabrication speed of ca 10 mm/s [65].

Nanoimprint lithography (NIL) is a low-cost nanopatterning technique for 2D and 3D structures, consisting on transferring a pattern from a mold to a surface [66]. This technique that can be carried out in three different ways: heating, ultra-violet curing (UV-NIL) and micro contact imprint. However, UV-NIL has the advantage to achieve higher resolutions than the other two techniques [76]. In UV-NIL, the mold is brought into contact with a wafer previously coated with photoresist and solidified with UV light. A resolution of 30 nm can be achieved [77].

Focused-ion beam nanolithography relies on a beam of ions to locally modify a surface coating, to mill a substrate or to deposit materials [67]. The resolution in milling surfaces is around 5–10 nm. One advantage of this technique over other techniques is that it can mill structures with irregular geometries and it requires few processing steps. One disadvantage is the slow milling speed of this serial technique. With focused-ion beam nanolithography it is also possible to deposit materials. The desired material to deposit is in the gas phase and it is let to adsorb on the surface. Afterwards, the ion beam decomposes the adsorbed molecules into a volatile component and a non-volatile component. The non-volatile component remains deposited on the surface. Minimum sizes achieved in deposition are in the range of few tens of nanometers [67]*.*

A more conventional technique is the electron beam (e-beam) nanolithography [68]. It uses an electron beam to etch a substrate surface locally or to modify locally a layer of a responsive polymer (e-beam resist) in order to obtain a pattern for further processing. Like focused-ion beam nanolithography, the e-beam nanolithography can also deposit materials at the nanoscale level [68].

Electrospinning is a technique that allows for the fabrication of a nanofiber-based meshwork. A solution with the desired polymer is ejected through a capillary towards a substrate by applying a high-voltage electric field. Long fibers with diameters in the range from 2 nm to several micrometers can be generated [69–72]. However, electrospinning has not been yet satisfactorily employed to obtain nanofibers from natural proteins such as fibronectin. Recently, an alternative method has been developed where nanofibers of extruded fibronectin through a nanoporous aluminum oxide membrane were obtained. This method is based on a mechanical force to provoke fibrillogenesis (generation of fibers) of fibronectin [78].

Nanoskiving is a less conventional technique for the fabrication of nanostructures, where basically a thin metal film is embedded between two epoxy layers. One of the epoxy layers contains a nano- or micropattern on which the metal layer is deposited (e.g., through vapor deposition). Then, a second epoxy block is cured on top of the deposited metal layer and the molding of this second epoxy layer represents the underlying nanostructure. Thus, thin sections (ca. 30 nm) can be obtained by ultramicrotomy, which are then transferred to a silicon substrate used as a master to mold silicon on top. Finally, by treating the epoxy–silicon sandwich with oxygen plasma the epoxy is eliminated, rendering the metal structure on the silicon substrate [46,73].

Colloidal lithography is a technique relying on the arrangement of colloid particles, on 2D or 3D surfaces, to use it as masks for subsequent etching or sputtering processes. It is a low-cost technique that does not require complex equipment since the pattern can be obtained by spin-coating the surface or by dipping it in the colloidal solution. Moreover, this technique allows for large surface patterning. The size of the colloids is tunable and determines the resolution of the pattern. Resolutions of few tens of nanometers can be achieved [74–75].

#### 1.4 Materials selection

The importance of choosing the appropriate material for cell–substrate interaction studies depends on the inherent ability of the material to be modified in its surface chemistry since biological cell adhesion via integrins or other adhesion molecules will generally not directly occur to inorganic or organic polymeric materials. Thus, further modification of the surface with adhesive molecules, for example with proteins from the extra cellular matrix is required. Additionally, it may be desirable to tune the mechanical stiffness of the material since recent advances in cellular mechanobiology have demonstrated the drastic effect of material compliance on various cell functions [79–82]. Moreover, the material of choice should also be suitable for the fabrication method used to generate micro- and nanoscale topographies. To be compliant with cell experiments the materials have to be implicitly non-cytotoxic in in vitro cell studies, or should be biocompatible for (future) in vivo applications [3–4,48,83–86].

There is a wide variety of materials including polymers, silicon, metals, ceramics, and composites made from various combinations of such materials [3,48,85–86]. Due to the vast amount of literature on different materials, only the most relevant materials used in studies with vascular cells are described below. Additional examples for materials are listed in Table 1 and in some other review articles [4–5,46–47,71,86–87]

Polymers from natural sources can be divided in either protein-based (e.g., collagen, fibrin, matrigel, elastin), polysaccharide-based (e.g., hyaluronic acid, chitin, agar, dextran, alginate) polymers, rubbers (e.g., *cis*-poly(isoprene), or polyesters (e.g., polyhydroxyalkanoates) [88–97]. Although they are typically non-cytotoxic and biocompatible, it is often very challenging to use them for most microfabrication processes. Independently of their use as pure or as combined polymeric materials, they frequently lack a clearly defined architecture, and they have a variable chemical composition and often complex mechanical properties. These disadvantages and difficulties in using natural polymers for the fabrication of cell culture substrates strongly motivated the development of alternative synthetic substrates [4,86–87,98–100]. Polymeric synthetic materials are the broadest and most diverse class of biomaterials available for cell research [89]. Some of these materials enable a good control of their surface chemistry, mechanical properties and geometry. Moreover, their non-cytotoxicity, their ease to use with many fabrication techniques and often the simplicity of their synthesis makes them to be widely used within the field of biomaterials [4–5,55,85,99–102]. Examples of the most representative synthetic polymeric materials used for vascular cell studies are poly(dimethylsiloxane) (PDMS) [6,61,103–106], poly(ethylene glycol) (PEG)-derived polymers [5,87,98,107–113] poly(acrylamide) (PAA) [50,114–116] and poly(lactic acid) (PLA) [117–118]. Alternative materials used for micro- and nanostructuring are glass [119]*,* ceramics [119–122] or natural polymers that can also be synthetically modified by, e.g., functionalizing with an artificial polymeric group [113,123]. Coating of these materials with silicon carbide, expanded polytetrafluoroethylene, tantalum, and hyaluronan has also been applied [124–127].

Apart from substrate topography, cells also respond to mechanical properties of the substrate and to the surface chemistry. Therefore, it is of importance to control these properties in order to precisely study, modulate or predict cell behavior. In the following section, a brief summary of the most common methods for control of the surface biochemistry and of the mechanical properties of the substrate are given.

#### 1.5 Surface (bio)functionalization

The surface (bio)chemistry of a material may regulate cell adhesion, survival, proliferation and differentiation of vascular cells or progenitor cells [10,31,126,128–131]. In order to make a biological meaningful contact with a surface, cellular trans-membrane adhesion molecules such as integrins need to interact with specific counterparts, generally ligand molecules of the extracellular matrix or molecules with similar motifs [132–136]. The interaction between cell-surface receptors and the substrate can be specific, where a cell ligand on the substrate specifically interacts with a cell receptor, or unspecific, where cell receptors interact unspecifically with the substrate due to electrostatic interactions. The sum of these ligand–receptor interactions, basically the biological adhesion, is signaling outside–inside and is a key factor for regulation of cell functions [137–138]. Therefore, adequate and controlled (bio)functionalizing of a materials surface is desired to have a predictable influence on cell behavior. Moreover, some synthetic materials are not promoting cell adhesion; a functionalization of the surface prior of cell contact becomes necessary to render it cell-adhesive.

There are numerous methods and strategies for chemical surface functionalization. It will be beyond the scope of this review to give a detailed account; only a few examples can be depicted. One simple and often used way to functionalize a surface non-specifically with molecules is by simple physisorption, often after enhancing the surface charge of hardly adhesive surfaces [139]. The latter is typically achieved by oxidizing the surface. Surface oxidation can be performed by means of oxygen plasma treatment, ultra violet (UV) light (for polymers) or chemical treatment [140–144]. Physisorption of molecules is typically an easy method for coating a surface more or less homogenously [139]. Apart from its simplicity, there is limited control and predictability about the number of adsorbed molecules, their orientation and three-dimensional configuration, and thus, their biological functionality. The sometimes relatively weak adhesion strength of the molecules is disadvantegous too. Using more advanced chemistry for immobilizing the molecules of interest in a controlled fashion [145–148].

Typical molecules used for non-specific surface coatings are poly-L-Lysine (PLL) and poly(lactic acid) (PLA), both interact unspecifically with cells through electrostatic interactions [149–150]. A more native coating of artificial surfaces can be achieved by absorbing molecules from the extracellular matrix (ECM). Commonly used cell ligands enabling a specific cell adhesion are either the full molecules or peptides with motifs from ECM molecules such as fibronectin, laminin, collagen and vitronectin [151–154]. One has to keep in mind, that in most in vitro and particularly in in vivo environments plenty of different proteins are getting in contact with the surface and alter the initial coating, thus making it difficult to maintain defined coating over longer time periods.

Control of the spatial distribution and density of molecules is for many biological investigations an interesting option. Such patterning with adhesive molecules can be realized by several techniques. Commonly used are microcontact printing [53,155] dry lift-off [58,156], dip-pen nanolithography [157–158], and block copolymer micelle nanolithography [159]. All these methods are just exemplary techniques allowing the (bio)chemical modification of structured surfaces for in vitro cell studies. Despite the precision of the modification that many of these methods achieve, it is important to keep in mind that in contact with a biological environment (e.g., cell culture media containing serum) there is an abundance of additional molecules, which may get absorbed to the surface of the substrate in an uncontrolled fashion. Additionally, cells can actively modify the surface biochemistry, for example by secreting ECM molecules or by rearranging the structure of the molecules attached to the surface, thus altering the initial surface chemistry drastically.

#### 1.6 Mechanical properties of micro- and nanostructured substrates

Cells can respond to changes in the mechanical properties of a substrate. Depending on the tissue in which cells live, the stiffness of the ECM can strongly vary from very low stiffness (e.g., brain: ca. 0.1–3 kPa) to intermediate stiffness (e.g., muscle: ca. 8–17 kPa) to high stiffness (e.g., cartilage: ca. 25–40 kPa) even reaching values in the order of megapascals or gigapascals, e.g., in bones [48,79,85–87,160]. In order to imitate in vitro the mechanical properties of the natural ECM, different materials are available that can be tuned in their mechanical properties. The most relevant and commonly used materials for this purpose are poly(ethyleneglycol) diacrylate (PEG-DA) or other PEG-derived polymers [5,87,98,107–113], poly(acrylamide) (PAA) [50,114–116], and poly(dimethylsiloxane) (PDMS) [6,61,103–106]. They are all not cytotoxic, relatively easy to handle, cheap to produce and their stiffness can be tuned over a wide range (from few tens of pascals to mega- or gigapascals). PEG-DA hydrogels consists of a polymer of ethylene glycol diacrylate monomers [48,161]. By varying the length of the polymer and its ratio with a crosslinker molecule the pore size of the hydrogel and thus its stiffness can be varied. Their elasticity can be tuned over a wide range from below 1 kPa to above 100 MPa [79]. An additional feature of PEG hydrogels is their low protein absorption. This characteristic makes them often to the coating material of choice when surfaces need to have low protein absorption [36,162–163]. Commonly used in cell culture experiments are PAA hydrogels [50,114–116]. They are made of acrylamide (the monomer), bis(acrylamide) (the crosslinker molecule) and a photoinitiator, which triggers polymerization. By varying the ratio between monomer, crosslinker and photoinitiator, as well as the intensity of UV light and the exposure time, the pore size of the hydrogel and consequently the elasticity can be tuned. Stiffness from PAA hydrogels can be tuned from ca. 50 Pa to more than 700 kPa [50,114–116]. Due to their easy availability and handling, PAA gels have found frequent application in cell mechanics studies [50,114–116,164–165]. PDMS is an elastomeric material [6,61,103–106]. Unlike PEG and PAA, PDMS is not a hydrogel but a hydrophobic polymer. Variation in hydrogel stiffness changes usually the meshwork properties and water content of the gel [114]. In the elastomer polymer, only the cross-linking density is varied. The stiffness of PDMS is modified by altering the ratio between monomer and curing agent, curing temperature, and curing time [114–115,166]. The Young’s modulus of PDMS can vary from 0.1 kPa [114] to a few megapascals [166]. All these three artificial polymeric materials are by themselves non-adhesive for cells. Therefore, surface functionalization with cell adhesive molecules is essential prior to cell culture studies.

In summary, the engineering of defined micro- and nanostructured cell culture substrates covers a whole range of different fabrication techniques and various materials. Generally, the fabrication methods applied to structure the surface of a (bulk) material limit the choice of materials (since not all materials are compatible with all fabrication techniques).

### 2 The vascular cell system and the responses of vascular cells to surface topographies in the micro- and nanometer range

The vascular system is one of the key systems of the human body and sustains normal human physiology during development, human life span and response to injuries [130]. Blood vessels are built from and regulated by an inner layer of vascular endothelial cells (ECs), and an outer layer of vascular smooth muscle cells (SMCs) (Figure 2) [167–168]. ECs are lining the inner part of the blood vessel (tunica intima), forming the so-called endothelium, and therefore they are in contact with the blood stream. Normally, ECs orient themselves to the direction of the blood stream. The endothelium acts as a barrier between the lumen of the vessel and the surrounding tissue, mediating for example leukocyte extravasation. It also plays a role in blood clotting and angiogenesis (formation of new vessels). On the other hand, SMCs are found in an outer layer surrounding the endothelium (tunica media). These cells are responsible for regulating the blood pressure by contracting or dilating. SMC contractility is chemically regulated by nerve cells and by ECs. The two vascular cell types are key players in vascular disease such as stroke, heart attack, and vascular occlusions [169–170]. A complete understanding of vascular cell biology requires a systematic, structural and separate analysis of the multiple interdependent signaling pathways in the cells types, the surrounding tissue and the blood [171–176]. Thereby, a multitude of stimulating signals, such as messenger molecules, ECM, pulsatile blood flow and endogenous electrical fields exist in and around the vasculature [177–179]. Additionally, in blood vessels of healthy humans, the regulation of SMC proliferation and migration is normally rigidly regulated by the endothelium formed by ECs [180].

Some important biological processes in vivo are mediated by signals that the features of the surface provide to adhering cells. For example, neuronal axons are guided by aligned Schwann cells, which at the same time are thought to be oriented by the ECM [181–182]. Recently, it has been demonstrated in mice that Schwann cells are guided by blood vessels [183]. In wound healing, another important biological process, cells are guided by the ECM to migrate towards the affected area for regeneration and healing [184].

**Figure 5:**
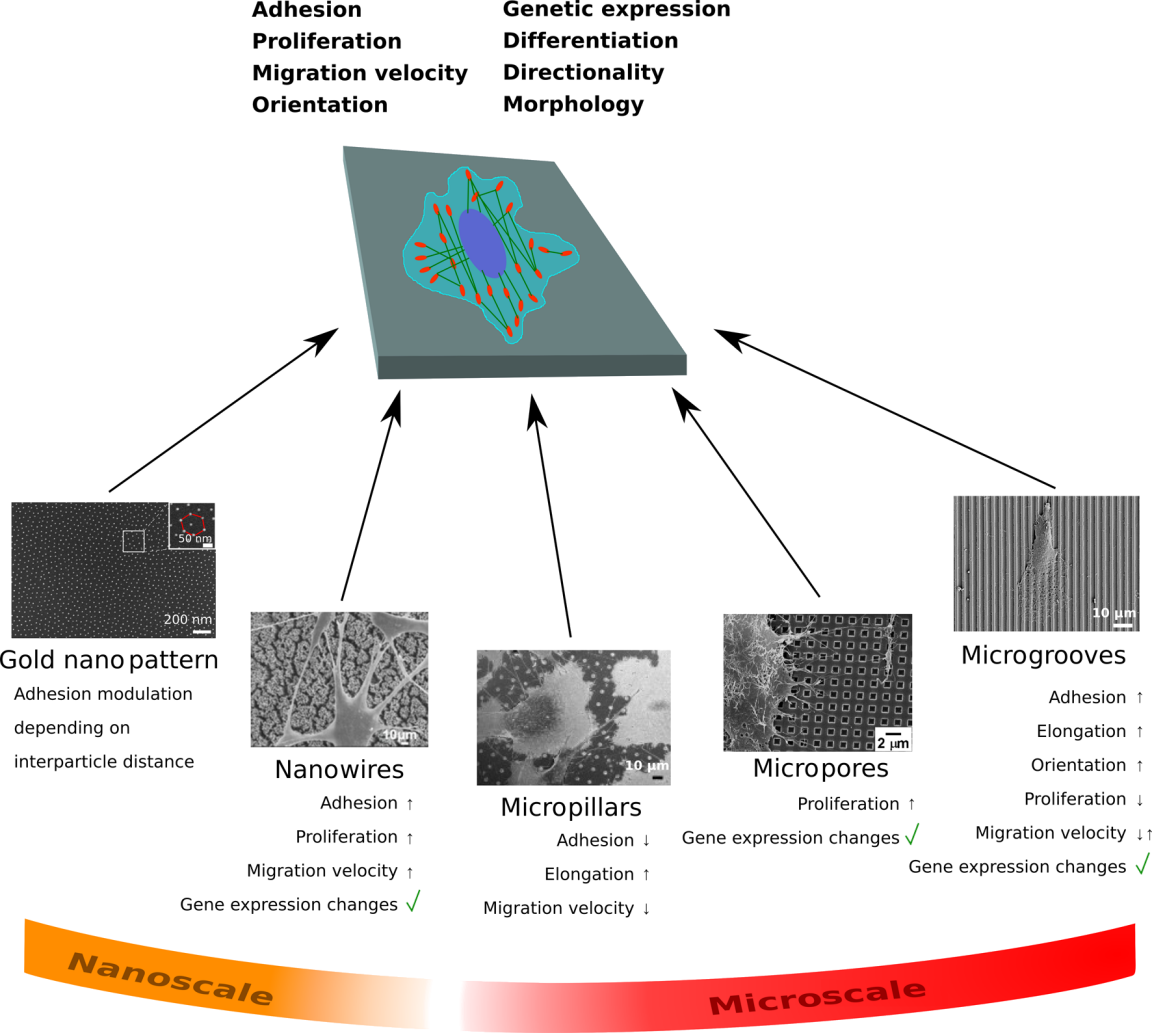
Endothelial and smooth muscle cells on surface topographies with different sizes (at the micro- and nanoscale) and geometries react by changing their adhesion strength, their proliferation rate, their genetic expression, by adapting their morphology, by migrating directionally, by changing their migration speed, or by inducing stem cell differentiation or cell reprogramming. Double arrow pointing up and down means that depending on the study or experimental conditions that parameter increases or decreases. The SEM image “Nanowires” has been reproduced with permission from [206], copyright 2014 the authors. SEM micrograph “Micropillars” has been reproduced with permission from [207], copyright 2015 Elsevier. The SEM image “Micropores” has been reproduced with permission from [208], copyright 2014 Elsevier.

To date, many studies have been carried out in vitro in order to elucidate the role of material properties in complex biological processes. It is known that micro- and nanoscale topographies of a substrate can influence cell adhesion, morphology, proliferation rate, migration velocity and directionality, gene expression, stem cell differentiation and even the epigenetic state of a cell (Figure 5) [10,12,79,185–197]. In vivo, the ECM where ECs and SMCs attach to, provide them micro- and nanotopographical stimuli to regulate their behavior [58]. Therefore, the in vitro investigation of cell type-specific functions and responses with vascular cells to surface topographies, either in the micrometer or in the nanometer range, provide new solutions to control the behavior of such cells and has already attained much interest [3,5,12,23–24,30–31,45,198]. Recent research has tried to elucidate mechanisms by which the independent stimulation of endothelial (ECs) and smooth muscle cells (SMCs) may be achieved by introducing surface topographies [8–12,199–200]. One of the main interests of these studies are the micro- and nanostructuring of medical implants for the in vivo control and stimulation of (vascular) cells behavior [8–10,12,201–202]. For example, it has been demonstrated that an efficient approach to improve the functions of a medical stent is the application of a morphological texture to the stent surface (topography), which controls and regulates the behavior of vascular cells [126,203]. Generally, the investigations of the cellular behavior upon culturing cells on structured surfaces have been performed with vascular cells from different species such as human, mouse, rat, and bovine and from different organs, for example, aorta, umbilical vein, bladder, and lung [5,16,21,23–25,204–205]. For a systematic overview of the information presented in the following sub-sections refer to Table 1.

#### 2.1 Survival and proliferation rate of vascular cells are influenced by surface topography

Live/dead staining is the most used assay to determine cell survival on a particular substrate and hence the cytotoxicity of a material is assessed [14,209]. Besides this live/dead cell investigation, biochemical proliferation assays, such as EdU or BrdU staining, are frequently applied to determine the cell division rates [26,204–205,210–211]. Often, the proliferation behavior of cells is simply evaluated by counting the number of cells and comparison of the actual number with the number of initially seeded cells [26,204–205,210–211].

**SMCs:** Proliferation of SMCs is differently regulated depending on the feature geometry and size. Some studies reported that proliferation of SMCs was negatively affected by PDMS microgrooves [204–205] and titanium oxide (TiO_2_) nanotube surfaces [31]. However, nanopits positively regulated SMC proliferation and gene expression [30]. In other studies, human SMCs from the vascular system and bladder, showed an increased proliferation rates on a poly(glycolic acid) (PGA) mesh, as well as on poly(ether urethane) (PU) and poly(lactic-*co*-glycolic acid) (PLGA) substrates with nanoroughness [41–42,44].

**ECs:** Similar to SMCs, the regulation of ECs proliferation depends also on the shape and size of topography. The proliferation rate of ECs has been reported to increase on metals (Ni, Ti and Co) and polymer substrates (PLGA and PCL) with nanoroughness present on the surface compared to the EC proliferation rate on flat surfaces [10,29,31,45]. Nevertheless, the proliferation rate of ECs on colloidal silica-coated surfaces with nanoroughness was decreased compared to ECs on flat surfaces [43].

For both types of vascular cells it is not clear through which mechanism the cell proliferation is influenced by the surface topography. Therefore, more research has still to be performed in these cell types to determine how substrate shape and feature dimensions correlate with cell proliferation.

#### 2.2 Structured surfaces influence the morphology, adhesion, and motility of vascular cells

Cells cultured on micro- and nanostructured substrates tend to change their morphology and their adhesion behavior/machinery according to the substrate topography (surface shape and size) as well as they frequently adapt their motility (Figure 4C) [5,13,209,212–215].

**ECs:** Previous studies showed a dependence of the strength of endothelial cell adhesion on the surface structure and its size. For example, ECs on nanoislands with low height (13 or 18 nm) showed an increased adhesion and spreading. However, on higher nanoislands (27 nm and above), the adhesion and spreading of ECs were reduced compared to those on flat surfaces [38–40]. In another study, ECs cultured on PEG nanopost structures showed stronger adhesion compared to those cultured on flat PEG substrate [36]. A different work demonstrated that ECs on silicon nanoposts, revealed stronger adhesion and spreading in comparison to ECs on flat silicon surfaces [210]. It has also been reported that the nanoroughness of metal (Ni, Ti and Co) and polymer (PLGA and PCL) surfaces improved EC adhesion compared to flat surfaces [10,29,31,45]. It is possible that the increase in EC adhesion is due to an increase in ECM protein adsorption and/or change of cell adhesions sites of these proteins probably caused by the increase of boundaries and surface energy on the surface [10,36].

Many cells show directed migration and a polarized morphology on nano- and microstructured substrates. The process by which cells orient and migrate along the longest axis of a surface feature is called contact guidance [186,216]. Many cell types on different surface topographies of various dimensions have been observed to experience contact guidance [217–222]. The most commonly used surface structure to study this phenomenon consists of arrays of ridges and grooves. Generally, cells orient preferably stronger along the direction of grooves and ridges, the narrower and deeper/higher these structures are [5,12,156,192,221–225]. It was observed that few tenths of nanometers in structure depth was already sufficient for some cell types to trigger contact guidance [201–202]. The limit of cell sensing, by filopodia, so far has been reported to be 10 nm-high nano-islands [226]. Furthermore, cell alignment to the direction of grooves was predicted with an automatic controller model [227]. This model concluded that cell alignment along the direction of grooves is proportional to the square of the aspect ratio (depth to width ratio) of the grooves/ridges [227]. As follows, some examples of morphological adaptation of vascular cells on substrate topographies are explained. For an extensive summary and literature review of vascular cell reactions to topography see Table 1.

**SMCs:** SMCs revealed on microgrooved PDMS substrates an enhanced aspect ratio (cell length to width ratio) and a parallel alignment of the cell body with respect to the groove axis (Figure 4C) [21,24]. Rat-derived SMCs aligned stronger along the direction of microgrooves, the narrower these grooves were [21]. Moreover, cells also change other morphological parameters, such as cell area or elongation, depending on surface structure shape and size [228]. For example, the elongation of SMCs was enhanced by the groove structure [204]. However, a structure composed of nanopits demonstrated no significant influence on SMC morphology [30].

**Figure 6:**
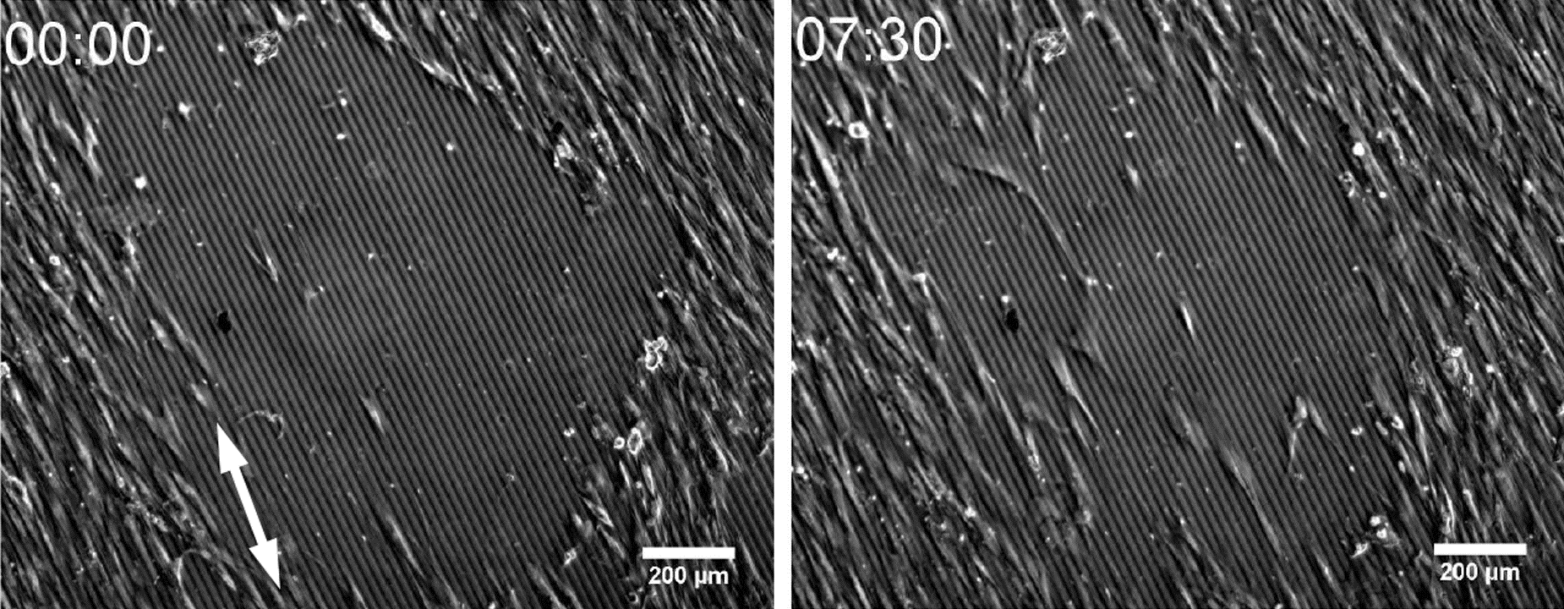
Human vascular smooth muscle cells (SMCs) cultured on a micrometer-sized grooved surface made of poly(dimethylsiloxane) (10 µm groove width; 650 nm groove depth). The exemplary images show the cell guidance reaction of the SMCs in a wound healing experiment. After 7 h 30 min the SMCs migrated preferentially along the direction of the micrometer-sized grooves.

**ECs and SMCs:** A similar effect was observed for SMCs, as well as ECs, cultured on nanogrooved structures, where the cells aligned and migrated in parallel with respect to the groove axis [15,25,204,229]. Internal cell structures important for topography detection and for conferring cell shape (i.e., focal adhesions (FAs) that are complexes of proteins anchoring to the substrate and regulating cell adhesion strength, and actin cytoskeleton) have been observed to change and adapt to surface topography. Probably the influence of the topography on these internal structures provokes the morphological change of the cells [204,215]. These morphological adaptations of the cells to surface structures were shown to be cell-type dependent [12]. This observation is important to consider in the design of cell type-specific medical implants since topographical cues of an implant could specifically and differently instruct cell reaction [12]. In addition to the tendency that cells orient their body along microstructures, cells also prefer to migrate along the longest axis of the structures present on the surface (Figure 6) [230–231]. In different studies, both SMCs and ECs, were observed to migrate directed along the groove direction [12–13,204,214,232–233]. It has been reported for some cell types that their migration velocity on microstructured surfaces increased compared to flat surfaces [212,234–237]. Nevertheless, there is still no clear consensus about the effect of surface topography on SMCs and ECs migration velocity [32,214,232–233,238]. A possible explanation to the differences in migration velocity on topographies would be the dynamicity of FAs. Dynamic FAs of ECs were correlated with a higher migration velocity than more stable FAs [213]. Moreover, the FA size was correlated with migration velocity. Generally, the bigger the FAs are, the faster cells migrate [239]. A different study found a correlation between cell stiffness and SMCs migration velocity. When cell stiffness increased, migration velocity decreased and vice versa. The increase of cell stiffness correlated with the increase in F-actin (filamentous actin) and vinculin (a protein from FAs) [240]. Nevertheless, systematic studies correlating the topography shape and size with vascular cell migration velocity need to be performed. Additionally, the cell mechanisms enabling directed migration to surface topography and influencing on migration velocity have still to be elucidated. These factors could have a great potential for the design of, e.g., stents [241].

#### 2.3 The cytoskeleton and cell-matrix adhesions of vascular cells cultured on micro/nanostructured surfaces

It is necessary to understand the signal pathways that transduce external physical stimuli into internal biological responses. The transduction of ECM surface topography information requires many intracellular mechano-sensitive elements and processes that finally lead to a cellular reaction (Figure 1 and Figure 2c) [173,175,242–243]. Often the signaling of several different mechano-sensors is combined and finally summed up. Thus, to be able to control cell adhesion and alignment in a cell-specific manner it is important to ask how a cell senses surface topography. The ECM physical signals can be transmitted through focal adhesions and the cytoskeleton system often by a signaling cascade initiated by integrin receptor activation [172,244–247]. Thus, one possible and likely scenario is the detection of the surface topography by cytoskeleton elements (in particular actin) and focal adhesions, and the probing of the topography by protrusions and filopodia (Figure 1 and Figure 2c) [226,248–250]. Thus, some studies analyzed intracellular structures such as the cytoskeleton and cell–substrate adhesion sites of cells cultured on nano/microstructured substrates in detail [13,15,20,213,215,251–252]. For example, the reorganization of the actin cytoskeleton has been observed in experiments where cells were placed on small ECM islands and then showed limited spreading [247].

**SMCs:** SMCs showed on microgrooved PDMS substrates a parallel alignment of actin filaments with respect to the groove axis [21,45]. In another study, focal adhesions of the same cell type grown on microgrooved substrates were more mature along the grooves, hence more tension was most likely created and cells aligned parallel being thus guided by microtopography [15]*.* In contrast to this work, some other studies claim that focal adhesions and actin stress fibers development are not necessary for contact guidance to take place [217–218]*.* In different studies, both human vascular and bladder SMCs, increased FAs size on a PGA mesh, and PU or PLGA nanoroughness substrates [41–42,44]. However, it is still not known if actin filaments are already polymerized along grooves or if their orientation is due to preferential actin contraction along grooves.

**ECs:** It was reported that the focal adhesion area from endothelial cells was increased on microgrooved substrate (groove dimensions: 1 µm depth, width) compared to the area of focal adhesions of cell cultured on flat substrate or shallower grooves [20]. Protein unfolding or conformation change at the boundaries between grooves and ridges, could facilitate FA formation and increase of its size [253].

**SMCs and ECs:** In an additional report, the spreading properties and focal adhesion system of EC and SMCs on nanopatterned ECM-mimicking surfaces was evaluated [251]. For this, the authors applied an array of biofunctionalized gold nanostructures. The gold nanoparticles on these surfaces have a diameter of 8 nm and had interparticle spacings of 40 nm or 90 nm and were conjugated with a RGD-peptide or a REDV peptide (R: arginine; G: glycine; E: glutamic acid; D: aspartic acid; V: valine) or with vascular endothelial cadherin (VE-cadherin) [251]. Non-functionalized surfaces or surfaces with spacing larger than 73 nm failed to induce the formation of FAs and actin stress fibers [251,254]. The universal distance-dependence for focal contact formation and cell adhesion shown previously for other cell types (e.g., MC3T3-osteoblasts, REF52-fibroblasts, 3T3-fibroblasts, and B16-melanocytes [251]) hold also true for the two vascular cell types (ECs and SMCs) investigated [255]. A distance-dependent behavior for ECs and SMCs on VE-cadherin decorated nanopatterns was also demonstrated here. Although both cell types adhere equally poor on VE-cadherin compared to the RGD and REDV peptides, a universally characteristic cell adhesion behavior depending on the ligand spacing was indicated [255].

#### 2.4 Signal transduction pathways and cell nuclei morphology of vascular cells cultivated on micro/nanostructured substrates

It has been observed in other studies, that the activation of many membrane proteins such as ion channels, membrane-associated G proteins coupled receptors (GPCR), and receptor tyrosine kinases (RTK) are related to ECM physical stimuli [256–264]. Surface topography also induces the activation of intracellular signaling molecules such as Rho GTPase, mitogen-activated protein kinases (MAPK), extracellular signal-regulated kinases (ERK), and Jun N-terminal kinase (JNK) [265–268]. Overall, mechano-transduction finally often leads to the activation of specific nuclear transcription factors, like the early growth response (Egr-1), the nuclear factor NFκB, and the activator protein (AP-1) [265–268].

**SMCs:** In case of SMCs, nanopits structures regulated their gene expression [197]. On titanium oxide (TiO_2_) nanotube surfaces, vascular SMCs expressed more SMC α-actin (a marker of differentiation) [129].

YAP/TAZ (Yes-associated protein/transcriptional coactivator with PDZ-binding motif) is a protein complex that induces the expression of proliferative genes. YAP/TAZ acts as a mechanical checkpoint and is not only regulated by the Hippo pathway but also by the reorganization of the actin cytoskeleton [247,269–270].

**ECs:** It has been demonstrated that ECs cultured on small areas coated with ECM protein (below 300 µm^2^) showed a weak actin stress fibers network (less actin bundles) and a switch of the YAP/TAZ localization from the nucleus to the cytoplasm compared to the fully spread cells [269]. A similar effect has also been observed for ECs growing on soft ECM substrate (ca. 0.7 kPa), where the actin cytoskeleton was weakened and the nuclear localization of YAP/TAZ was reduced, compared to the cells culture on a fibronectin-coated glass surface. The protein Rho and the formation of actin stress fibers, but not actin polymerization alone, were required to increase YAP/TAZ nuclear localization [269].

Nuclei also tend to adapt their morphology directly to surface microtopography in a similar manner as cell bodies adapt [17,271]. However, in some cases the orientation of nuclei differs to that of the cell body [211,272–275].

**SMCs:** SMCs showed on microgrooved PDMS substrates a parallel alignment of cell nuclei with respect to the groove axis [21,24].

The fact that the nuclear shape is influenced by microstructures leads to the assumption that some changes in the genetic material could take place. It was revealed that changes in nuclear shape, non-invasively induced by microgrooves, caused reorganization of nuclear lamina and chromosomes repositioning [276]. Some different gene regulations were attributed to these changes in chromosomes positions [276]. In a different work, a dramatic drop of SMC proliferation on micropillars was reported and was argued that the deformation of the nuclear lamin was responsible of this change in proliferation rate [277].

**ECs:** Differently regulated genes from cells on microstructures, in comparison to cells on flat surfaces, showed that endothelial cells on microgrooves down-regulated genes related to the cell cycle as well as their gene for β1 integrin [278].

In the future, it would be interesting to study the influence of nucleus morphology in vascular cells gene expression and the role of mechanotransduction mechanisms involving gene regulation. Moreover, a study correlating actin-mediated cell tension, with nuclei deformation and genetic expression changes would be of interest, since it was previously found a relation between actin-mediated cell stiffness and nucleus deformation [279].

## Conclusion

In this review article, we have presented the state of the art of the most commonly used materials and methods to micro- and nanostructure surfaces for vascular cell investigations. Moreover, vascular cell responses to these topographical stimuli were also presented and discussed. Although many studies, with both cell types (ECs and SMCs), have shown the influence of material, geometry and size of topographical features, on cell morphology, migration, and proliferation, there is not yet a correlation between different geometries and sizes of topography and cell response. For example, there is no clear consensus between structure dimensions and migration speed for both vascular cells.

In order to better understand how these cell responses change depending on the surface topography, the main internal structures playing a role in cell mechanotransduction (focal adhesions and actin cytoskeleton) have been studied. FAs and actin cytoskeleton were commonly observed in many studies to orient along structures as cells do. Although some studies correlated differences in FA size and dynamicity with cell migration speed, further research has still to be done in order to broaden the observation. Another important aspect that it should be addressed in future research is how FAs and actin cytoskeleton are influenced by the topography.

To further deepen in the understanding of vascular cell behavior on topographies, studies have been performed to analyze gene and protein expression. Changes in genetic expression attributed to a morphological change in cell nucleus have been shown. However, the relation between shape of the nucleus and gene expression levels is still not known yet. In fact, the protein complex YAP/TAZ, was observed to regulate cell proliferation on ECs seeded on substrates of different stiffness. Since the actin cytoskeleton is responsible of reducing cell proliferation through YAP/TAZ phosphorylation, it would be interesting to investigate if surface topography affects YAP/TAZ activation through changes in the actin cytoskeleton.

Although a lot of investigations on vascular cells reactions to surface topographies are still to be done, this research will eventually lead to a better understanding of important biological processes (e.g., tissue regeneration) and to the development of new medical implants such as stents with modified chemical, mechanical and topographical properties. These new medical implants will enable the in vivo control the behavior of vascular cells without using, e.g., pharmacological substances.
